# Autoreactive T cells and thymic atrophy pathway in thymic hyperplasia patients with myasthenia gravis

**DOI:** 10.3389/fneur.2026.1744302

**Published:** 2026-03-23

**Authors:** Xuemin Wang, Pei Chen, Wenjing Yang, Jiaxin Chen, Shiyin Li, Chuanming Luo, Xin Huang

**Affiliations:** 1 The Seventh Affiliated Hospital of Sun Yat-sen University, Shenzhen, China; 2 The First Affiliated Hospital of Sun Yat-sen University, Guangzhou, China; 3 Guangxi Hospital Division of the First Affiliated Hospital of Sun Yat-sen University, Nanning, China

**Keywords:** autoreactive T cells, forkhead box N1, myasthenia gravis, thymic atrophy pathway, thymic hyperplasia, WNT family member 4

## Abstract

**Background:**

T cells play a vital role in antigen presentation, immune tolerance imbalance, and secretion of pathogenic cytokines in the progression of Myasthenia Gravis (MG). Previous studies have founded that patients with thymic hyperplasia accompanied by MG exhibit autoreactive T cells in thymus that can recognize self-antigens at the neuromuscular junction (NMJ), leading to the destruction of acetylcholine receptors (AChR) and the appearance of muscle weakness. Furthermore, Thymic hyperplasia is associated with T-cell-related autoimmune diseases, and increased expression of WNT4 and FOXN1 are beneficial to the growth of thymic cells and the maintenance of the thymic microenvironment.

**Methods:**

The samples were obtained from surgical patients who met the criteria, and this study was ethically approved. We first measured the proportion of T cell subsets in the peripheral blood and thymus of thymic hyperplasia patients with and without myasthenia gravis. Then, we quantified the WNT4 and FOXN1 proteins in the thymus. Finally, we used the Pearson correlation test to analyze the correlation between autoreactive T cells and the thymic atrophy pathway.

**Results:**

Flow cytometry clarified that Th1 and Th17 cells were elevated in the peripheral blood and thymus of patients with MG (1.207 ± 1.444 and 3.788 ± 0.6920 in Th1, 7.683 ± 1.025 and 0.3127 ± 0.1936 in Th17). Western blotting revealed increased expression of key molecules in the thymic atrophy pathway, WNT4 and FOXN1, in the MG group. Additionally, the abnormal activation of the thymic atrophy pathway may be one of the causes of autoreactive T cell production.

**Conclusion:**

Our study has identified an increased number of Th1 and Th17 cells in the peripheral blood and thymus of patients with thymic hyperplasia associated MG. Quantitative analysis of proteins and RNA indicates that the expression of WNT4 and FOXN1 is upregulated in patients with thymic hyperplasia accompanied by MG. Additionally, the abnormal activation of the thymic atrophy pathway may be one of the causes of autoreactive T cell production. This research provides a potential perspective on the pathogenesis of MG in patients with thymic hyperplasia.

## Introduction

Myasthenia gravis (MG) is the most common and specific autoimmune disease, characterized by self-reaction T cells and autoantibodies against the acetylcholine receptors (AChRs), including abnormal activation of T cells (particularly helper T cells) leading to erroneous recognition of self-antigens (1–3). Thymic hyperplasia refers to the pathological condition characterized by the presence of lymphoid follicular hyperplasia or true parenchymal hyperplasia within the thymic tissue (4). Thymic hyperplasia is often accompanied by abnormalities in T-cell immune tolerance mechanisms, one of the pathogenic mechanism through which thymic hyperplasia contributes to MG (4, 5). Thymic hyperplasia (particularly the formation of germinal centers) disrupts autoimmune tolerance through “pathogenic T-B cell collaboration within the thymus,” presenting self-antigens (such as the acetylcholine receptor) to naïve T cells and supporting their activation and differentiation into helper T cells, thereby driving B cells to locally produce AChR antibodies within the thymus, ultimately leading to MG (6).

Thymic atrophy is a cascading process initiated by age-dependent functional degeneration of thymic epithelial cells. Its essence lies in the infiltration of adipose tissue replacing thymic parenchyma, leading to a progressive loss of the thymic microenvironment’s ability to support T-cell development. Infections, inflammation, and other conditions can also accelerate the process of thymic atrophy (7, 8). This is a complex biological process involving multiple factors and pathways. Its core is the decline in the functional maintenance network of thymic epithelial cells (centered on FOXN1 and WNT4), which is precisely regulated by signaling pathways such as the hormonal environment (sex hormones, GH/IGF-1), chronic inflammation (NF-κB, NLRP3), and metabolism/adipogenesis (PPARγ) (9). In early-onset, AChR antibody-positive MG patients, the thymus not only fails to undergo physiological atrophy but instead maintains activity or undergoes hypertrophy due to persistent autoimmune inflammation (primarily associated with the IL-23/Th17 pathway) and lymphoid hyperplasia (formation of germinal centers) (3, 10). Its essence lies in the breakdown of pathogenic immune tolerance within the thymus, transforming it into a functional “autoantibody factory.” Driven by pro-inflammatory factors, ectopic germinal centers similar to lymph nodes appear within the thymus. These structures support the activation, maturation, and class switching of B cells, leading to the direct and abundant production of AChR antibodies within the thymus (5). This active lymphoid hyperplasia physically and functionally counteracts the process of thymic atrophy. These processes collectively indicate that abnormal pathways of thymic atrophy exist in the thymus of MG patients.

WNT4 and FOXN1 are the core master transcription factor for the development, maturation, and maintenance of thymic epithelial cells (TECs), directly determining whether the thymic microenvironment can support T-cell generation (10). Reactivation of FOXN1 can promote the reconstruction of the thymic microenvironment (11). By maintaining the function of medullary thymic epithelial cells (particularly AIRE expression) and facilitating negative selection, FOXN1 contributes to the clearance of self-reactive T cells. Deficiency in its function may lead to the escape of autoimmune T cells (12). As an early signal for thymic organogenesis and epithelial progenitor differentiation, WNT4 indirectly provides the foundation for establishing central immune tolerance by ensuring normal development of thymic structure and microenvironment, lead to the dysregulation of the thymic immune tolerance (13).

Hence, we hypothesize that abnormalities in the thymic atrophy pathway could be one of the pathogenic factors leading to the occurrence of MG in thymic hyperplasia patients. The elucidation of this mechanism helps to further clarify the role of thymus in the pathogenesis of MG, expands the related research of MG, and establishes a theoretical basis for the development of specific immunotherapy methods to inhibit the abnormal activation of T cells in MG.

## Methods

### Ethical approval and acquisition of human thymic samples

We separate human thymus samples into two groups (control and MG), which were obtained from patients undergoing heart surgery or thymectomy at the First Affiliated Hospital of Sun Yat-sen University, with the ethical approval from Sun Yat-sen University. Control group patients were screened based on the following criteria: (1) Absence of autoimmune diseases. (2) Lack of severe coronary heart disease, hypertension, diabetes, tuberculosis, and serious conditions like tumors. (3) No acute infections in the past. (4) Those who have not undergone any other immunotherapy using corticosteroids or immunosuppressants. (5) Thymectomy was performed due to other reasons. MG group was screened based on the following criteria: (1) Typical clinical characteristics of muscle fatigue, positive neostigmine test, electromyography support: positive low-frequency RNS and/or SPEMG. (2) Positive serum AChR-Ab. (3) Those who have not undergone treatment using corticosteroids or immunosuppressants. (4) Excluding other concomitant autoimmune diseases like hyperthyroidism, systemic lupus erythematosus, Sjogren’s syndrome, rheumatoid arthritis and serious conditions like coronary heart disease, hypertension, diabetes, tuberculosis, tumors, etc. (5) Excluding those with any acute infections in the month prior to surgery. (6) MG patients who are clinically evaluated to need thymectomy and confirmed to hav**e** thymic hyperplasia post-surgery. A total of 9 thymus samples from the HC group and 10 thymus samples from the MG group were collected (Table 1). The thymus samples for our HC group were obtained from patients with congenital heart disease, who were relatively young. Additionally, the thymus tissues we were able to acquire from older individuals within the intended HC control cohort were largely replaced by fat (adipose tissue) and thus unsuitable for subsequent research. Consequently, an age mismatch does exist in our study design, which is acknowledged that is a limitation of our research. Pathological examination revealed the presence of hyperplastic lymphoid follicles in all 10 thymus tissues from the MG group, while germinal centers (Gcs) are hallmark structures of hyperplastic lymphoid follicles (14).

**Table 1:** The list about informations of thymus samples in the experiment.

Participant	Gender	Age	AChR antibody titer (nmol/L)	Osserman classification	Hyperplastic lymphoid follicles
HC1	F	19	−	−	−
HC2	F	21	−	−	−
HC3	M	19	−	−	−
HC4	M	23	−	−	−
HC5	M	18	−	−	−
HC6	F	26	−	−	−
HC7	M	22	−	−	−
HC8	F	25	−	−	−
HC9	F	20	−	−	−
MG1	F	46	0.89	II B	+
MG2	F	37	0.57	II B	+
MG3	M	51	0.65	II B	+
MG4	F	49	0.71	II B	+
MG5	M	39	0.62	II B	+
MG6	F	38	0.49	II B	+
MG7	F	53	0.44	II B	+
MG8	M	29	0.69	II B	+
MG9	F	58	0.71	II B	+
MG10	F	40	0.96	II B	+

### Reparation of single-cell suspensions of lymphocytes from peripheral blood and thymus

Peripheral blood samples (Supplementary Table 1) from thymic hyperplasia patients obtained from the outpatient department or operating room are mixed evenly in a 1:1 ratio with 1X PBS and transferred into a 15 mL centrifuge tube, layered over 2 mL of Ficoll. After 20 min of centrifugation at 800 g with acceleration and deceleration settings at 2 and 0, respectively, the lymphocyte white ring layer need be transferred into a new centrifuge tube and added to 10 mL of PBS for centrifugation for 10 min. This washing step is repeated twice. The resulting cell pellet is cultured with complete medium (RPMI medium containing 10% Fetal Calf Serum, 1% glutamic acid, 0.1% mercaptoethanol, and 1% pen/strep) for subsequent experiments, or the cells are cryopreserved in a gradient freeze at −80°using cryopreservative (10% DMSO and 90% Fetal Calf Serum). The patient’s thymus obtained from the operating room is prepared into a single-cell suspension by grinding, enzymatic digestion, and filtering. Resuspend tissue remnants in 10 mL of fresh Collagenase/DNase solution plus 10 mL of RPMI plain and add Trypsin/EDTA (1:50 from 2.5% stock) in the solution. Then Incubate at 37 °C for 40 min with gentle rotation. During the last 10–15 min of the incubation add FCS (1:5) to neutralize the activity of Trypsin. Afterwards, centrifuge at 110 x g for 2 min at RT to sediment the tissue fragments. Collect the cell supernatant and discard the sedimented tissue fragments and centrifuge the cell supernatant at 400 x g for 10 min. Next, aspirate supernatant and resuspend cell pellet in RPMI complete. Subsequently, count viable cells using a hemocytometer. Ultimately, lymphocytes are enriched (15).

### Flow cytometry analysis

To analyze the proportion changes of Th1 and Th17 cells in human peripheral blood and thymus, we revived the frozen cells and added 2 mL medium complete for overnight incubation to restore the cell morphology and activity. The next day, we added the lymphocyte stimulant (BD Biosciences, Le Pont de Claix, France) at a 1:100 ratio and cultured it under 37 °conditions for 8 h. Finally, staining was performed according to the cytokine flow cytometry staining procedure. Multi-color flow cytometric analysis was performed using the following fluorochrome-labelled monoclonal anti-human antibodies: anti-CD4 Alexa Flour 700 (317,426, biolegend), anti-IFN-γPE/Cyaine7 (502,528, biolegend), anti-IL17A APC (512,334, biolegend). Cells were measured with a CytoFlex S flow cytometer (Beckman Coulter, Brea, CA, USA). CytExpert 2.0 software (Beckman Coulter, Brea, CA, USA) was used to analyze the raw flow cytometry data.

### Western blotting

Cellular proteins were extracted using RIPA lysis buffer (Thermo Fisher Scientific, Waltham, MA, USA) supplemented with protease and phosphatase inhibitors (Thermo Fisher Scientific, USA). Total protein concentration was determined with a BCA assay kit (Thermo Fisher Scientific, USA) following the manufacturer’s instructions. Equal amounts of total protein (30 μg per lane) were separated by electrophoresis on 10% SDS-PAGE gels and transferred onto 0.45 μm pore-size polyvinylidene difluoride (PVDF) membranes (Merck Millipore, USA) using a wet transfer system. The membranes were blocked with 5% non-fat milk for 1 h at room temperature and subsequently incubated overnight at 4 °C with the following primary antibodies: FOXN1 (diluted 1:2000, Proteintech), WNT4 (diluted 1:2000, Proteintech), GAPDH (diluted 1:5000, CST). Protein bands were visualized by Clinx Chemiluminescence Instrument at equal exposure time. After extensive washing, the membranes were probed with horseradish peroxidase (HRP)-conjugated anti-rabbit secondary antibody (1:2000, Thermo Fisher Scientific, USA) for 1 h at room temperature. Protein bands were detected using Immobilon Western Chemiluminescent HRP Substrate (Merck Millipore, USA) and visualized with an ImageQuant LAS 4000 imaging system (GE Healthcare Life Sciences, Chicago, USA). Protein expression levels were normalized to GAPDH and quantified using ImageJ software.

### RNA extraction and real-time quantitative PCR (RT-qPCR)

TRIzol reagent (Invitrogen) was used to extract RNA from thymus. After quantifying RNA with Nanodrop 2000, cDNA was reverse-transcribed from 1,000 ng total RNA using PrimerScript RT Master Mix (RR036A, Takara) and diluted at 1:50 with DEPC water. The RT-qPCR was performed utilizing a SYBR Green PCR kit (TaKaRa; Dalian, China) in the Roche Light Cycler 480 System. The results were normalized to the mRNA expression of GAPDH in each sample, and gene expression was calculated using the 2 − ΔΔCT method. Primer sequences (forward and reverse, respectively) are given in as follow (Table 2).

**Table 2:** List of the RT-qPCR primers used in the study.

Gene	Forward	Reverse
GAPDH	GGAGCGAGATCCCTCCAAAAT	GGCTGTTGTCATACTTCTCATGG
FOXN1	AAATGCCCTGTCCCTAGCTC	CTCAGTTGCCCCAGAAACGAA
WNT4	GAGGAGACGTGCGAGAAACT	TACTGGCACTCCTCAATGGC

### Statistical analysis

The GraphPad Prism 10 application (GraphPad Software, La Jolla, CA) was utilized for graph construction and statistical analyses. Datas are analysed as mean (MG - HC) ± standard deviation (SD). Our data passed the normality test through histograms and exhibited a normal distribution. Statistical differences were assessed using t-test. We used histograms to visually illustrate the changes between the relative expression of WNT4 and FOXN1 and the number of Th1 and Th17 cells in HC group and MG group. *p* < 0.05 was considered statistically significant (Table 1).

## Results

### Th1 and Th17 cells increase in the peripheral blood in MG patients

**Figure 1:**
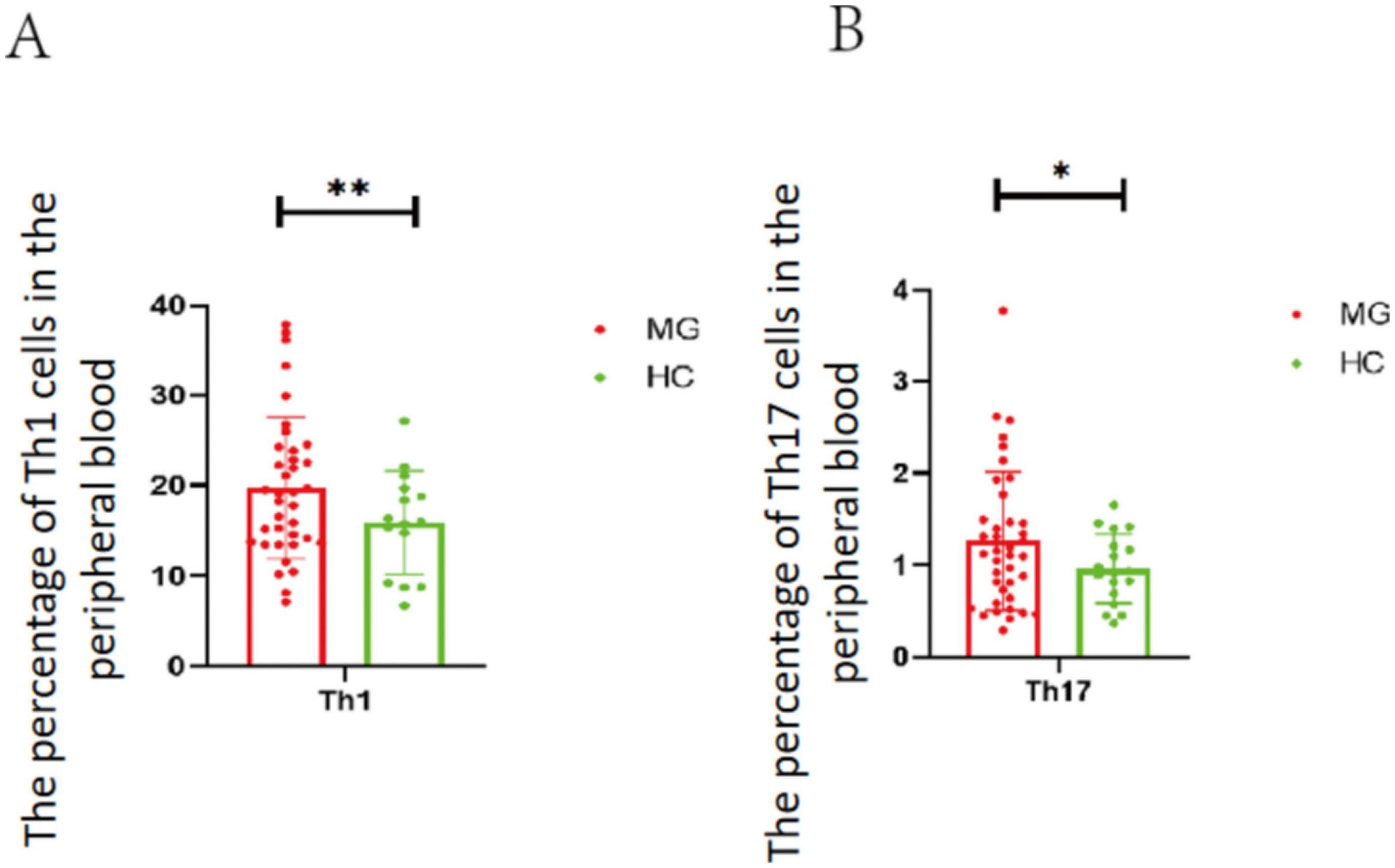
The number of Th1 and T17 cells in the peripheral blood of the HC and MG groups. **(A)** The number of Th1 cells in the peripheral blood of the HC and MG groups. **(B)** The number of Th17 cells in the peripheral blood of the HC and MG groups. 17 cases in the HC group and 39 patients in the MG group. Data were tested for normality and *p* values between the two groups were analyzed using the *t*-test. * *p* < 0.05, ** *p* < 0.01.

We conducted experiments on T cell subsets in the peripheral blood of these patients. Compared with the healthy control group, the results from flow cytometry analysis indicate that the number of Th1 and Th17 cells are elevated in the peripheral blood of patients with MG (Figures 1A,B), which *p*-value is less than 0.05 with the mean difference between the healthy control group and the experimental group is 1.207 ± 1.444 and 0.3127 ± 0.1936.

### Th1 and Th17 cells increase in thymus of thymic hyperplasia patients with MG

**Figure 2:**
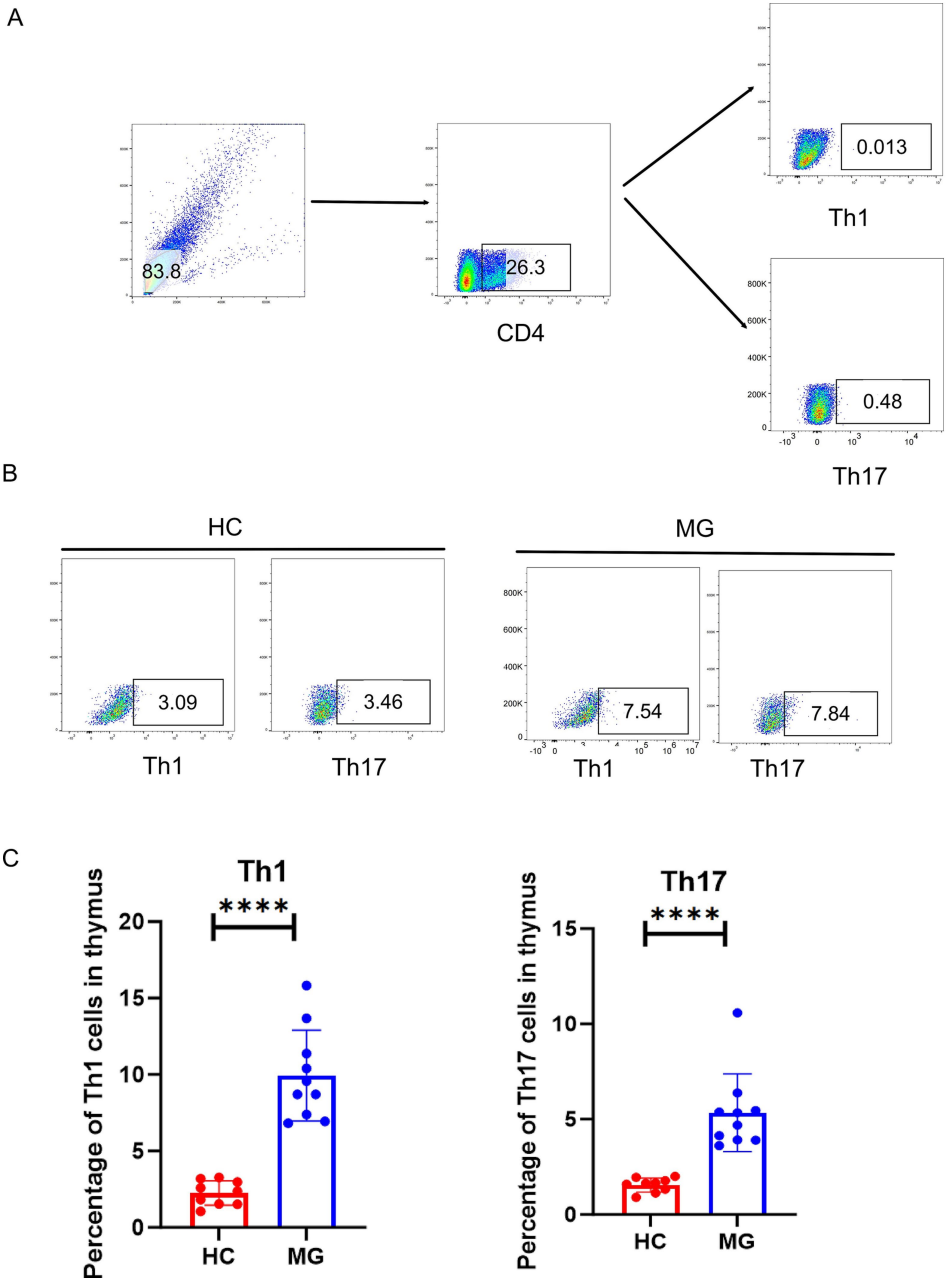
The flow cytometry results of Th1 and Th17 cells in thymus of the HC and MG groups. **(A)** The gating methods of flow cytometry. **(B)** The flow cytometry results of Th1 and Th17 cells in thymus of the HC and MG groups. **(C)** The number of Th1 and Th17 cells in thymus of the HC and MG groups. 10 cases in the HC group and 9 patients in the MG group. Data were tested for normality and *p* values between the two groups were analyzed using the *t*-test. **** *p* < 0.0001.

In order to confirm whether thymic hyperplasia patients with MG accompany the production of pathogenic T cells, we conducted flow cytometry analysis on Th1 and Th17 cells in the thymus (Figure 2B). In contrast to the healthy control group, our research has demonstrated that the proportion of Th1 and Th17 cells in the thymus of patients with thymic hyperplasia accompanied by MG significantly increase (Figures 2C, *P* < 0.0001). Respectively, the mean difference of Th1 and Th17 cells between the HC group and the MG group is 7.683 ± 1.025 and 3.788 ± 0.6920.

### The thymic atrophy pathway is associated with the onset of thymic hyperplasia patients with MG

**Figure 3:**
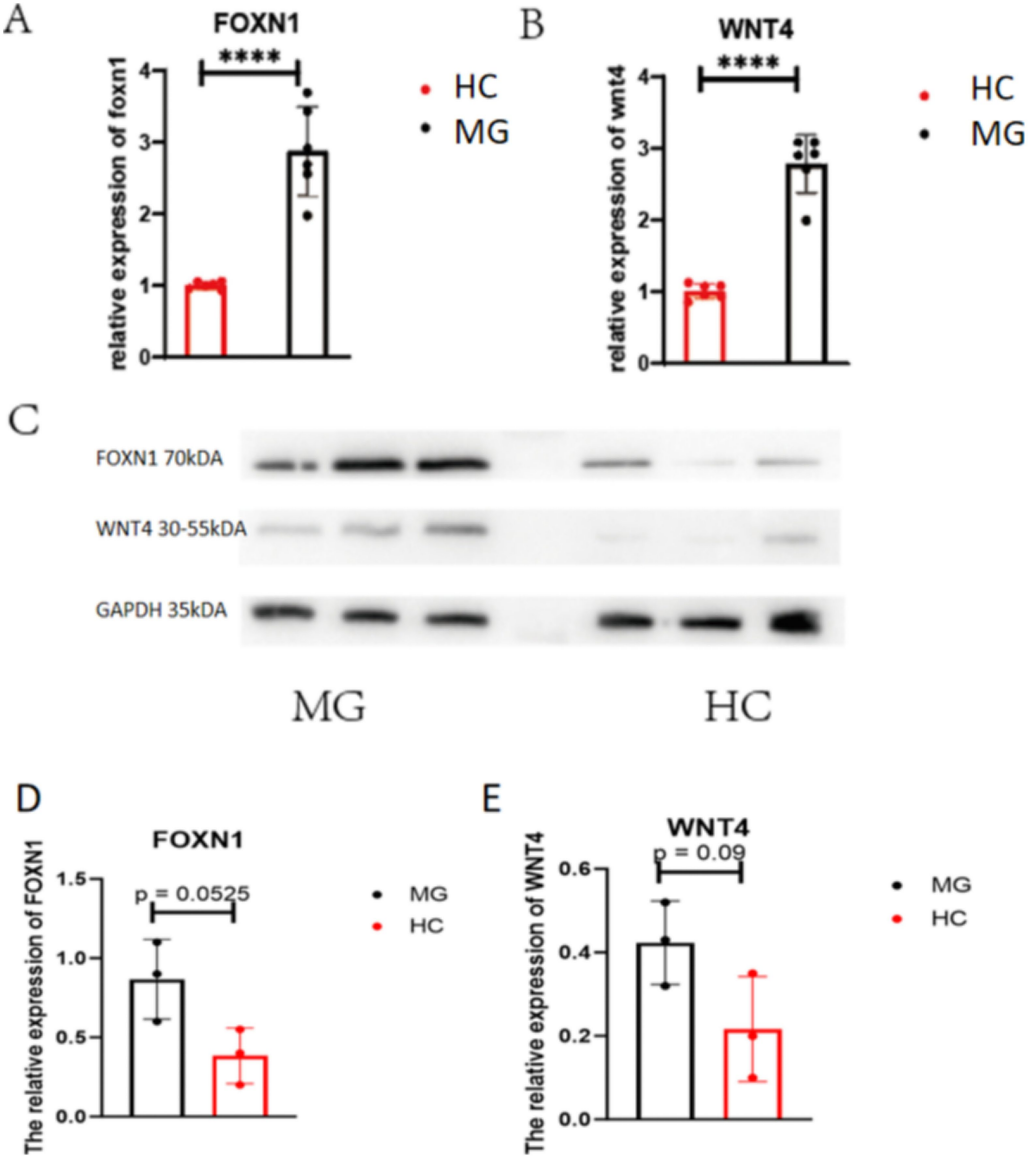
The relative expression of RNA and protein of WNT4 and FOXN1 in the HC and MG groups. **(A)** The relative expression of RNA of FOXN1 in the HC and MG groups. **(B)** The relative expression of RNA of WNT4 in the HC and MG groups. **(C)** The images about western blotting’s analysis of WNT4 and FOXN1 in the HC and MG groups. **(D)** The relative expression of protein of FOXN1 in the HC and MG groups. **(E)** The relative expression of protein of WNT4 in the HC and MG groups. Six cases in the HC group and 6 patients in the MG groups. Data were tested for normality and *p* values between the two groups were analyzed using the *t*-test. ****p < 0.0001.

Thymic epithelial cells (TECs) play a crucial role in thymocyte development, and their numbers determine the thymic capacity for lymphocyte production. With age-related thymic involution, TEC numbers gradually decline. However, in patients with MG, the thymus does not fully follow the physiological pattern of age-related atrophy; instead, it exhibits relative hyperplasia compared with age matched controls. Studies have shown that upregulating the expression of WNT4 and FOXN1 can promote thymic redevelopment. We therefore hypothesize that abnormalities in the normal atrophy pathway of the thymus in MG patients may contribute to the pathogenesis of the disease (10, 12, 16–18). We examined the protein and RNA expressions in thymus of two pivotal molecules in thymic atrophy pathway, WNT4 and FOXN1. The mean difference of WNT4 and FOXN1 between HC group and MG group in our research is 1.876 ± 0.2544 and 1.781 ± 0.1731, which results illustrated the upregulated relative expression levels of both molecules in the patient group with MG (Figures 3A,B, *P* <  0.0001). Images from protein blotting presented a notable increase in the expression levels of FOXN1 and WNT4 in the MG group (Figures 3C–E).

### The relative expression of RNA of WNT4 and FOXN1 and the number of Th1 and Th17 are elevated in thymic hyperplasia patients with MG

**Figure 4:**
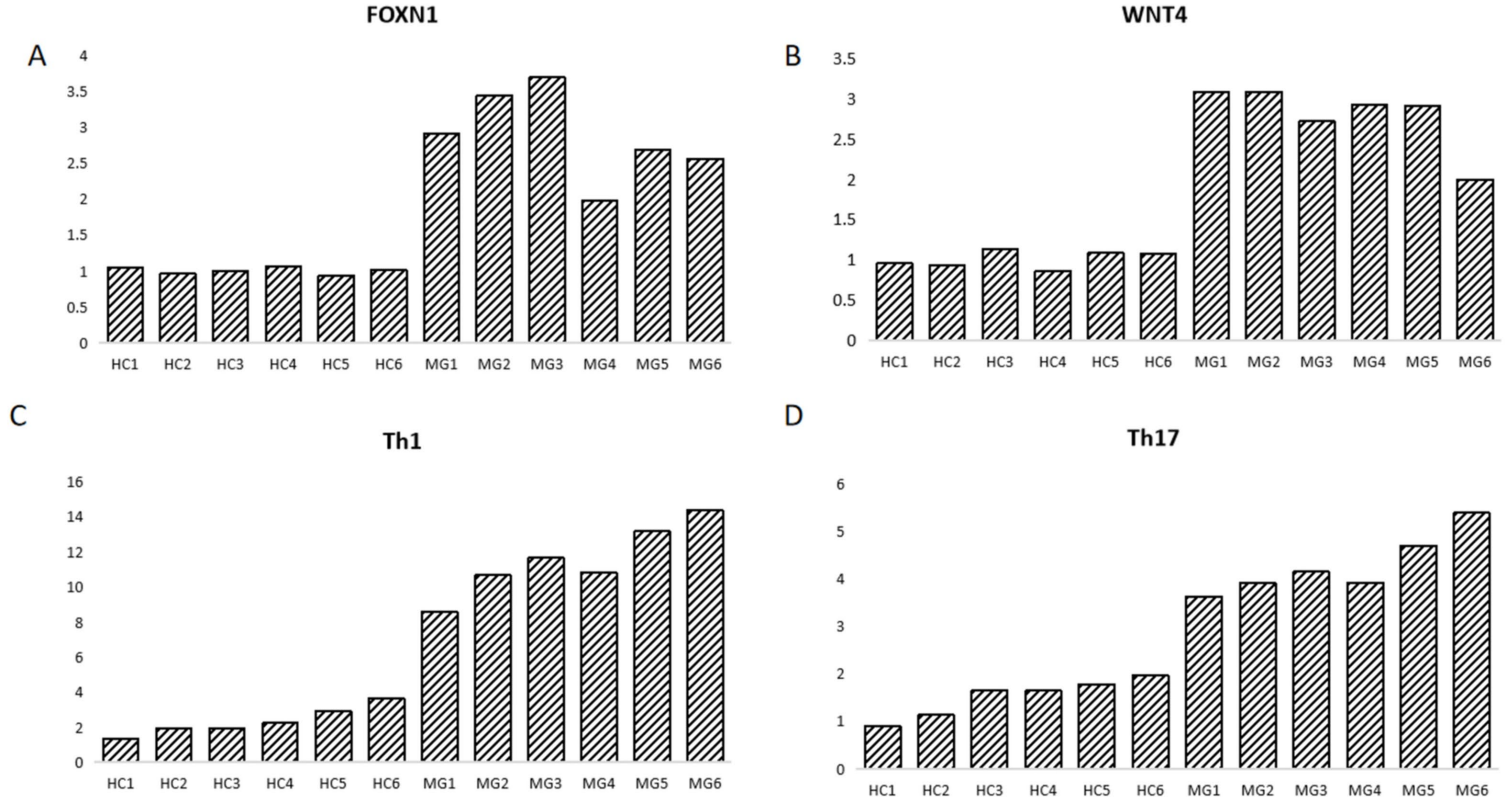
The relative expression of RNA of WNT4 and FOXN1 and the number of Th1 and Th17 cells in the HC and MG groups. **(A)** The relative expression of RNA of WNT4 in the HC and MG groups. **(B)** The relative expression of RNA of FOXN1 in the HC and MG groups. **(C)** The number of Th1 cell in the HC and MG groups. **(D)** The number of Th17 cells in the HC and MG groups. Six cases in the HC group and 6 patients in the MG group.

To visually demonstrate the changes the relative expression of RNA of WNT4 and FOXN1 and the number of Th1 and Th17 in the HC and MG groups, we employed histograms to assess the relative expression of these two proteins and the differences in these two cell populations between the HC group and MG group. Our results revealed that both the relative expression of RNA of WNT4 and FOXN1 and the number of Th1 and Th17 cells were elevated in thymic hyperplasia patients with MG (Figure 4).

## Discussion

In this research, we observed an increase in Th1 and Th17 cells in the blood and thymus of patients with MG-associated thymic hyperplasia. These changes were correlated with the abnormal activation of the WNT4/FOXN1-mediated thymic atrophy pathway. Therefore, we hypothesize that the aberrant expression of WNT4 and FOXN1 in the thymus of adult individuals contributes to the generation of pathogenic T cells, thereby triggering autoimmune diseases. Since the roles of WNT4 and FOXN1 in thymic hyperplasia patients remain unclear, our future research will employ animal experiments to determine whether overexpression of WNT4/FOXN1 can promote the production of autoimmune T cells.

MG is a autoimmune disease characterized by self-reaction T cells and autoantibodies against the acetylcholine receptors (AChRs), including abnormal activation of T cells (particularly helper T cells) leading to erroneous recognition of self-antigens (1–3). Th1 and Th17 cells collaboratively drive and amplify the autoimmune attack against the NMJ in MG. Th1 cells dominate classical pro-inflammatory signaling, while Th17 cells bridge inflammation and autoantibody production. Together, they disrupt immune tolerance through an imbalanced cytokine network and assist B cells in producing pathogenic antibodies through the secretion of pro-inflammatory cytokines in MG (3, 15, 19, 20). Similarly, our flow cytometry results indicate that in MG thymic hyperplasia, Th1 cells and Th17 cells are elevated in the peripheral blood, when compared to the HC group.

Thymic hyperplasia refers to the pathological condition characterized by the presence of lymphoid follicular hyperplasia or true parenchymal hyperplasia within the thymic tissue (5, 21, 22). Thymic hyperplasia creates a local microenvironment through aberrant signaling pathways and chemokine secretion(e.g., IFN-*γ*, IL17A), which facilitates the recognition of self-antigens (e.g., the acetylcholine receptor) by autoreactive T and B cells, promotes their abnormal activation, proliferation, and interaction, thereby breaking immune tolerance and initiating a systemic autoimmune response against target organs (4, 21). Therefore, the mechanism by which thymic hyperplasia leads to autoimmune diseases may involve impaired immune tolerance of autoreactive T cells within the thymus. In our study, an elevated proportion of Th1 cells and Th17 cells was observed in the thymus, when compared to the HC group, which may be associated with abnormalities in the thymic microenvironment of thymic hyperplasia. Immature thymic epithelial cells (TECs) may lead to the exposure of MHC II self-antigens, resulting in impaired T-cell immune tolerance and failure to effectively eliminate autoreactive T cells such as Th1 cells and Th17 cells.

Thymic atrophy is a multi-pathway regulated tissue remodeling process driven by the functional degradation and adipocyte infiltration of TECs, occurring with aging or under pathological conditions. This ultimately leads to the disruption of the thymic microenvironment, reduced output of naïve T cells, and immune function decline. In conditions such as infection, chemotherapy, radiation, or chronic diseases, elevated levels of inflammatory factors like TNF-*α* and IFN-*γ*, as well as increased glucocorticoids, can directly induce TEC apoptosis and suppress their function, resulting in thymic atrophy (12, 23). Numerous studies have demonstrated the involvement of WNT4 and FOXN1 in the development of T-cell autoimmunity. However, the role of thymic atrophy pathway in thymic hyperplasia remains understudied.

FOXN1 is a core transcription factor essential for maintaining thymic epithelial cell (TEC) function, and WNT4 regulates the expression of FOXN1. Together, they form a central regulatory axis that maintains thymic structure and function by controlling TEC proliferation, differentiation, and microenvironment organization. Reduced expression of these molecules leads to a decline in TEC numbers, impaired T-cell production, and disruption of immune tolerance within the thymic microenvironment. Conversely, reactivation of these two molecules in the thymus can promote thymic regeneration and development (11, 24). However, in patients with MG, the thymus often does not exhibit atrophy and is instead associated with the production of autoreactive T cells and autoantibodies. Therefore, we hypothesize that dysregulation of the thymic atrophy pathway may be one of the mechanisms by which thymic hyperplasia contributes to the development of myasthenia gravis.

Existing research has found that TECs are extremely sensitive to the expression levels of FOXN1. Normal FOXN1 expression maintains the postnatal microenvironment, and premature downregulation of FOXN1 can lead to a rapid involution-like phenotype, while overexpression can cause thymic hyperplasia and delayed involution of thymus (25). FOXN1 affects the expression of MHC II on TECs, thereby influencing the formation of T cell tolerance (9). WNT4 has been demonstrated to upregulate the expression of FOXN1 in the thymus (26). In the past researches, increased expression of WNT4 can inhibit the upregulation of markers linked to adipose differentiation and aging, indicating a significant function of WNT4 in the aging of the thymus (27, 28). Our results revealed that both the relative expression of WNT4 and FOXN1 and the number of Th1 and Th17 cells were elevated in thymic hyperplasia patients with MG. This trend may suggest that in thymic hyperplasia patients with MG, the abnormal activation of the thymic atrophy pathway is accompanied by an increase in autoreactive T cells. However, our study did not further elucidate the underlying mechanism between WNT4 and FOXN1 and Th1 and Th17 cells, which is a very important direction for our future research.

Previous studies have extensively elucidated the mechanisms of the thymic atrophy pathway in both thymic development and physiological atrophy. The decrease expression of WNT4 and FOXN1 induce thymic dysfunction, contributing to generation of autoimmune T cells has also been investigated (12, 24). However, the mechanism by which aberrant thymic development leads to MG has been scarcely explored. Our prior research found that MG patients are often associated with aberrant thymic development. Through further investigation, we discovered elevated expression of the key molecules of the thymic atrophy pathway in the thymus of patients with thymic hyperplasia accompanied by MG. Therefore, our study provides a novel perspective on the mechanism by which abnormal activation of the thymic atrophy pathway contributes to the occurrence of MG in patients with thymic hyperplasia.

Our study has certain limitations that should be noted. First, thymic hyperplasia patients and healthy controls were recruited from a single institution, necessitating further multi-center research. Moreover, our study did not establish a causal relationship between the thymic atrophy pathway and the two cell types. Therefore, large-scale, long-term research is needed to confirm the relationship between thymic atrophy pathways and autoimmune T cells. In future studies, we will elucidate the mechanistic interactions between WNT4 and FOXN1 and Th1 and Th17 cells employing both *in vivo* and *in vitro* experiments.

## Conclusion

Our study has identified an increased number of Th1 and Th17 cells in the peripheral blood and thymus of patients with thymic hyperplasia associated MG. Quantitative analysis of proteins and RNA indicates that the expression of WNT4 and FOXN1 is upregulated in patients with thymic hyperplasia accompanied by MG. Additonally, the abnormal activation of the thymic atrophy pathway may be one of the causes of autoreactive T cell production. This research provides a potential perspective on the pathogenesis of MG in patients with thymic hyperplasia.

## Data Availability

The original contributions presented in the study are included in the article/Supplementary material, further inquiries can be directed to the corresponding author/s.
