# Supplementary material for: Autoreactive T cells and thymic atrophy pathway in thymic hyperplasia patients with myasthenia gravis
Source: Front Neurol. 2026 Mar 23;17:1744302. doi: 10.3389/fneur.2026.1744302 (PMC13050652; doi:10.3389/fneur.2026.1744302)
Supplement: Supplementary file 1 [file Data_Sheet_1.pdf]

Supplement Table1 The list about informations of the peripheral blood samples in the experiment

	Gender	Age		Gender	Age
MG1	M	25	HC1	M	21
MG2	F	26	HC2	M	32
MG3	M	32	HC3	F	34
MG4	F	35	HC4	M	26
MG5	F	31	HC5	M	26
MG6	F	27	HC6	F	19
MG7	M	27	HC7	F	33
MG8	M	44	HC8	F	39
MG9	M	50	HC9	F	37
MG10	M	46	HC10	F	42
MG11	M	37	HC11	M	56
MG12	M	33	HC12	M	58
MG13	M	27	HC13	M	51
MG14	F	34	HC14	F	49
MG15	F	31	HC15	M	31
MG16	F	28	HC16	M	33
MG17	F	24	HC17	F	29
MG28	F	33			
MG29	M	35			
MG30	M	37			
MG31	M	32			
MG32	M	41			
MG33	M	46			
MG34	M	43			
MG35	F	52			
MG36	F	55			
MG37	M	57			
MG38	M	31			
MG39	M	34			
